# Revisiting the growth rate hypothesis: Towards a holistic stoichiometric understanding of growth

**DOI:** 10.1111/ele.14096

**Published:** 2022-09-11

**Authors:** Jana Isanta‐Navarro, Clay Prater, Logan M. Peoples, Irakli Loladze, Tin Phan, Punidan D. Jeyasingh, Matthew J. Church, Yang Kuang, James J. Elser

**Affiliations:** ^1^ Flathead Lake Biological Station University of Montana Polson Montana USA; ^2^ Department of Integrative Biology University of Oklahoma Stillwater Oklahoma USA; ^3^ Bryan College of Health Sciences, Lincoln, NE, USA and School of Mathematical & Statistical Sciences Arizona State University Tempe Arizona USA; ^4^ Division of Theoretical Biology and Biophysics Los Alamos National Laboratory Los Alamos New Mexico USA; ^5^ School of Life Sciences Arizona State University Tempe Arizona USA; ^6^ Department of Biology Lund University Lund Sweden

**Keywords:** carbon, ecological stoichiometry, growth rate hypothesis, nitrogen, phosphorus, protein, RNA

## Abstract

The growth rate hypothesis (GRH) posits that variation in organismal stoichiometry (C:P and N:P ratios) is driven by growth‐dependent allocation of P to ribosomal RNA. The GRH has found broad but not uniform support in studies across diverse biota and habitats. We synthesise information on how and why the tripartite growth‐RNA‐P relationship predicted by the GRH may be uncoupled and outline paths for both theoretical and empirical work needed to broaden the working domain of the GRH. We found strong support for growth to RNA (*r* ^2^ = 0.59) and RNA‐P to P (*r* ^2^ = 0.63) relationships across taxa, but growth to P relationships were relatively weaker (*r* ^2^ = 0.09). Together, the GRH was supported in ~50% of studies. Mechanisms behind GRH uncoupling were diverse but could generally be attributed to physiological (P accumulation in non‐RNA pools, inactive ribosomes, translation elongation rates and protein turnover rates), ecological (limitation by resources other than P), and evolutionary (adaptation to different nutrient supply regimes) causes. These factors should be accounted for in empirical tests of the GRH and formalised mathematically to facilitate a predictive understanding of growth.

## Graphical abstract

The growth rate hypothesis (GRH) posits that variation in organismal stoichiometry (C:P and N:P ratios) is driven by growth‐dependent allocation of P to ribosomal RNA. The GRH has found broad but not uniform support in studies across diverse biota and habitats. We synthesize information on how and why the tripartite growth‐RNA‐P relationship predicted by the GRH may be uncoupled and outline paths for both theoretical and empirical work needed to broaden the working domain of the GRH.

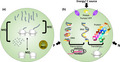

## INTRODUCTION

What biological mechanisms link the biochemical properties of cells to dynamical processes in ecosystems? Seeking answers to this question has led to the development of stoichiometric theory. Building on fundamental concepts of mass balance and established elemental frameworks (Lotka, 1922; Redfield, 1934; Reiners, 1986; Sprengel, 1828; von Liebig, 1855), Ecological Stoichiometry was first developed to explain how the balance of multiple elements shapes ecological interactions (Sterner & Elser, 2002). This approach has provided insight into how the elemental composition (in terms of carbon [C], nitrogen [N] and phosphorus [P]) of organisms impacts trophic dynamics and biogeochemical cycling. Biological Stoichiometry was developed as a complementary framework to explain sources of variation in organismal C:N:P stoichiometry in biochemical, cellular and evolutionary terms (Elser et al., 2006; Jeyasingh et al., 2014). Combined, the emergence of this stoichiometric approach has been accelerated by collaboration among biologists and mathematicians because principles of mass conservation make biological processes readily approachable in mathematical terms (Andersen et al., 2004; Elser et al., 2012; Kuang et al., 2004; Loladze et al., 2000; Peace et al., 2021).

**FIGURE 1:**
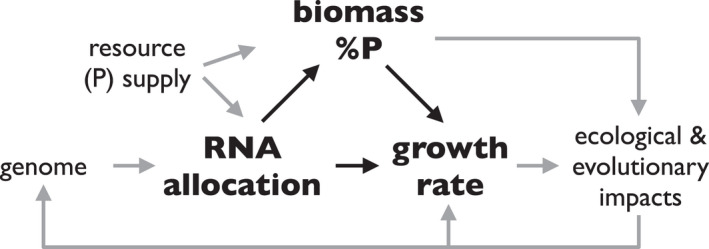
A schematic diagram of the growth rate hypothesis (GRH). Tripartite relationships that constitute the GRH (black arrows) are shown linking organismal %P, growth rate and RNA allocation, as well as potential ecological, evolutionary and genomic drivers and consequences of that coupling (grey). Note that a focus is placed on P content as it is most often the primary driver of variation in organismal C:P and N:P ratios (rather than variation in %C or %N).

The C:N:P composition of an organism shapes its ecological interactions across scales. At the ecosystem scale, biomass C:N:P stoichiometry determines the elemental composition of detritus generated from that biomass (Killingbeck, 1996), the rates and ratios of nutrients recycled by consumers (Caron et al., 1988; Elser & Urabe, 1999; Goldman et al., 1987a), as well as carbon use efficiency across scales from organisms to ecosystems (Cebrian & Lartigue, 2004; Goldman et al., 1987b; Manzoni et al., 2018). Given the ecological importance of stoichiometric variation, considerable effort has been expended in documenting and explaining differences in C:N:P ratios at the organismal scale. For example, an early focus in stoichiometric plankton ecology was given to explain the contrast of *Daphnia* (C:N:P ~ 80:13:1 molar) and calanoid copepods (~240:39:1) that was associated with food‐web driven effects on ecosystem N‐ vs. P‐limitation (Sterner et al., 1992). Since variation in zooplankton %P (i.e., P content expressed as a percentage of dry mass) can be up to 10‐fold higher than variability in %C and % N (Andersen & Hessen, 1991; Elser et al., 1996; Sterner & Elser, 2002), an understanding of the basis of variation and regulation of P content became an essential target of stoichiometric studies (Sterner & Elser, 2002). Towards that end, a key emphasis in Biological Stoichiometry has been the growth rate hypothesis (GRH), which posits a tripartite association (Figure 1) among P content (and thus C:P and N:P stoichiometry), allocation to P‐rich ribosomal RNA, and growth rate (Elser et al., 2000).

Over 20 years after its formal introduction (Elser et al., 2000), the GRH has stimulated a tremendous amount of research into the elemental underpinnings of growth. Here, we outline the working domain of the GRH and its original assumptions and discuss the biological insights it has generated. We also explore the cellular processes and environmental conditions responsible for deviations from GRH predictions. By doing so, we seek to clarify some of the confusion that exists around the GRH and outline important processes affecting GRH couplings that should be taken into consideration in the future. Finally, we discuss how mathematicians and empiricists can work together towards developing and implementing a robust and more inclusive GRH.

## DEVELOPMENT OF THE GRH

The GRH states that variation in organismal stoichiometry (in particular, C:P and N:P ratios) is driven by growth‐dependent P allocation to ribosomal RNA (Figure 1). To grow, organisms must translate C and N‐rich proteins through the use of P‐rich ribosomal RNA, meaning that growth rate is a primary trait governing organismal C:P and N:P stoichiometry. Note that in the following we focus on a variation on P content (rather than C:P or N:P per se), given that C and N contents show more modest variation than P content (González et al., 2017) and thus P is the primary driver of variation in organismal C:P and N:P ratios in most, but not all, situations.

While growth rate/RNA relationships were established in the 1950s (Kjelgaard et al., 1958; Schaechter et al., 1958), Sterner and Hessen (1994) first hypothesised the linkages between growth, RNA content and P content in zooplankton. This idea was further developed by Elser et al. (1996), which sought to describe how cellular biochemistry, life history and evolutionary differences in growth rate explained differences in the N:P stoichiometry of organisms. These ideas were codified in Elser et al. (2000), which extended this framework to a broader domain of biota and identified plausible genetic mechanisms, related to ribosomal RNA gene transcription, responsible for this tripartite coupling.

As originally set forth, the GRH was built on a set of key assumptions linked to the central core of organismal growth—the role of P‐rich ribosomes in protein synthesis.

Assumption A1: **RNA‐P.** Growth‐dependent variation in P allocation to RNA is proportionally large enough to drive changes in organismal P content.

Assumption A2: **Ribosome Allocation**. The number of ribosomes in a cell controls the overall rate of protein synthesis.

Assumption A3: **Constant Translation Rate**. Ribosomes translate proteins at or near their maximum capacity.

Assumption A4: **Constant Protein Retention**. A fixed fraction of synthesised protein accumulates in the cell, contributing to growth.

The GRH was explicitly formulated to apply to biota without major storage mechanisms of P (i.e., excluding vertebrates with P‐rich bones), which could undermine A1 by weakening relationships between organismal RNA and body P allocation (Elser et al., 2000). The GRH was also intended to entail growth variation that is *not* driven by temperature. Changes in temperature can alter growth rates; for example, high temperatures can allow enzymes and ribosomes to operate faster (undermining A3), potentially decreasing or leaving unchanged (as opposed to increasing) organismal N and P contents in support of translation at a given growth rate. Shortly after it was proposed, it became clear that the GRH may not be applicable for relatively large invertebrates (e.g., those >1 mg dry mass), as RNA contribution to total body %P is inversely related to body size (undermining A1; Gillooly et al., 2005). While there is a clear need to integrate all of these factors into stoichiometric theory (Cross et al., 2015), empirical work has not yet been conducted across a wide‐enough range of taxa for the development of holistic generalised models to proceed. As a step towards this goal and bearing these assumptions in mind, in the following we identify four mechanisms directly affecting organismal growth‐biochemical‐elemental coupling and outline an integrative research framework that will advance a predictive understanding of growth.

## FUNDAMENTAL MECHANISMS THAT AFFECT THE GRH

**FIGURE 2:**
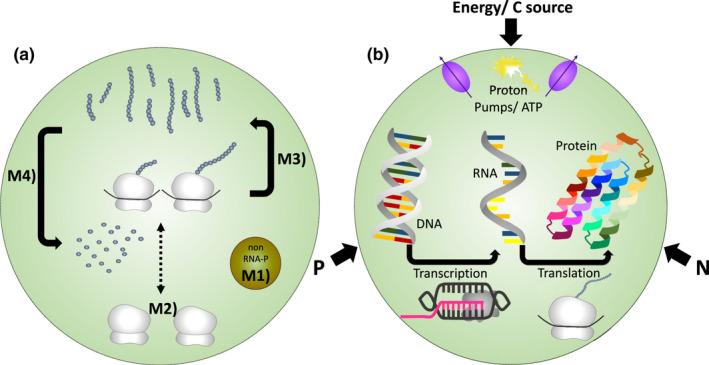
(a) A schematic depiction of four mechanisms that can influence the coupling of growth, RNA and P proposed by the GRH. On the organismal scale, the first mechanism (M1) involves contributions from pools of non‐RNA P, including storage of P in molecules such as polyphosphates. Mechanisms M2, M3 and M4 constitute changes on the molecular scale that individually or collectively affect the net protein production rate per ribosome. Mechanism (M2) involves change of the fraction of inactive ribosomes among all ribosomes. Mechanism (M3) entails differences in ribosome translation elongation rate and mechanism (M4) highlights protein degradation or protein turnover rate. (b) Effects of C‐, N‐ and P‐limitation on cellular functions. The figure shows the relationships between environmental resource supplies and cellular functions that influence growth rate and could result in deviations from the GRH under different types of resource limitation.

Because there is only one predominant metabolic pathway for protein anabolism (Ramakrishnan, 2002) and protein synthesis is a primary driver of growth (Milo & Phillips, 2015), strong RNA/growth relationships must generally hold in protoplasmic chemistry although the consistency of growth/P and RNA/P relationships are likely to be less reliable. Indeed, we propose that one or more fundamental mechanisms can weaken the tripartite associations posited by the GRH at the organismal scale. Figure 2a depicts these mechanisms in order of their corresponding assumptions (A1‐4); they are non‐RNA P‐storage (Mechanism M1), the active state of ribosomes (M2), translation elongation rate (M3) and protein turnover rate (M4). It is worth noting that many other environmental factors (e.g., temperature) and intracellular parameters (e.g., transcription) absent from this list can *indirectly* affect the couplings proposed by the GRH (hereafter, GRH decoupling) by influencing one or more of these four mechanisms. Therefore, by combining modelling approaches and empirical measurements of mechanisms M1‐M4, we can better understand the reasons why the GRH is supported or not, enabling us to better predict growth and biomass stoichiometry under ecologically relevant conditions.

Due to the empirical difficulty of measuring mechanisms M2‐M4, most of our understanding of GRH decoupling is related to M1, the influence of non‐RNA P storage. A significant but variable proportion of cellular P can be found in RNA, meaning that non‐RNA P found in storage or structural pools can decouple growth‐P‐RNA relationships by reducing the relative contribution of RNA‐bound P to the total P pool (Flynn et al., 2010). Perhaps the most well‐known example of P storage is inorganic polyphosphate, which is an ancient energy‐P molecule, found in cells from microbes to humans (Kulaev & Vagabov, 1983). Polyphosphate is most notably stored in vacuolar granules in microbes where it can contribute between 3 and 30% of total P (Bellinger et al., 2014; Deinema et al., 1985), explaining at least some of the wide variation in microbial stoichiometry recorded in the literature. Another potentially important P pool is phospholipid‐P, which can contribute up to 25% of microbial P (Van Mooy & Fredricks, 2010). The primary role of phospholipid‐P is that of a structural molecule forming the lipid bilayer of cell membranes, but this P may be released for metabolic use under severe P‐limitation (Van Mooy & Fredricks, 2010). Release of P from polyphosphate or phospholipids can interfere with GRH predictions in microbes transitioning from high‐ to low‐P conditions, as P is internally re‐allocated from storage or structural forms into metabolically active pools such as RNA with little or no change in overall organismal P content (Li et al., 2019; Martin et al., 2014). Accumulation of these molecules could also decouple growth/RNA relationships from P under other forms of nutrient limitation (discussed below), especially in natural microbial assemblages (Kornberg et al., 1999; Mullan et al., 2002).

Even in small invertebrates where the GRH would be expected to apply (i.e., organisms <1 mg dry weight; Gillooly et al., 2005), non‐RNA P pools can still obscure growth/P relationships. For example, insects can convert organic P into longer‐term storage in inorganic P‐Mg‐Ca granules inside their gut Malpighian tubules (Maddrell, 1971). Rivalling the P storage capacities of vertebrates, some crustaceans use a similar organ, the hepatopancreas, to store P reabsorbed from their Ca‐P‐based exoskeletons before moulting (Luquet & Marin, 2004). Large increases in crustacean carapace P content are observed during carapace formation (Sather, 1967), as P is secreted into the inner matrix of the carapace to bind Ca. These examples highlight the dynamic elemental interactions and multiple functions of P in storage, structure and metabolism in addition to those considered by the GRH. Our appreciation of the extent of non‐RNA P pools across the tree of life is far greater today than it was during the initial development of the GRH. These new insights require that non‐RNA P pools be incorporated in stoichiometric frameworks or risk further restricting their applicability. For example, 14%–35% of body P in juvenile *Daphnia* can be found in the carapace (He & Wang, 2020; Vrede et al., 1999), indicating that, in addition to microbial taxa, one of the two zooplankton genera that inspired the development of the GRH may fall on the edge of the hypothesis' original domain.

As the GRH is centred around P, mechanisms M1‐M4 can result in findings contradictory to the GRH under growth limitation by resources other than P (Elser et al., 2003). For example, in a study of N‐limitation in four marine phytoplankton species, growth rate was positively coupled to RNA content as predicted by the GRH, but RNA and P contents were *negatively* related due to storage of surplus P (M1; Liefer et al., 2019). A decoupling of growth from RNA and P contents can occur in *Daphnia* feeding across N‐limitation gradients (Elser et al., 2003), with animals retaining high %P and %RNA despite reduced growth. This may be explained by reduced translation elongation rates of abundant ribosomes under N‐limitation (M3), caused by temporary pauses in mRNA translation (known as “ribosome stalling”) due to reduced availability of N‐rich amino acids (i.e., glutamine; Li et al., 2018). Alternately, N‐ and C‐limitation can decouple growth rate from RNA and P in *Escherichia coli* through the accumulation of inactive ribosomes (M2, ~70% of the total ribosome pool; Li et al., 2018). An increase in inactive ribosomes weakens GRH relationships since these ribosomes contribute to the RNA‐P pool without producing proteins (and hence new biomass). While protein turnover rates (M4*)* of *E. coli* are similar under most forms of nutrient limitation (Nath & Koch, 1971), these rates can be much higher in the photoautotrophic bacterium *Synechococcus* under N‐ and sulphur (S)‐limitation, compared to P‐stressed conditions, due to the high requirements of N and S for constructing light‐harvesting pigments (Collier & Grossman, 1992). High rates of protein turnover keep proteins from accumulating in cells, thus decoupling growth from RNA and P. These examples suggest that systematically exploring the mechanisms that influence the GRH under different forms of nutrient limitation across different biota will be a promising avenue of future work.

In addition to these four mechanisms that affect the predictions of the GRH directly by invalidating its assumptions, the role of transcription has been a relatively neglected part of stoichiometric theory due to the low contribution of mRNA to the total RNA pool (~ 4% in mammalian cells, Wu et al., 2014; ~ 8% in bacteria, Levinthal et al., 1962). Ribosomes consist of both protein and RNA, meaning that elemental costs of transcription and translation could result in trade‐offs in ribosome production and abundance under different forms of resource limitation (Figure 2b; Kafri et al., 2016; Weiße et al., 2015). For example, the high P demands of transcription (three P atoms for each transcribed codon) can limit the transcriptional production of new ribosomal RNA in low‐P environments (Li et al., 2018; Loladze, 2019; Loladze & Elser, 2011). Similarly, translation ultimately depends on N availability (1‐to‐4 N atoms for each amino acid). Since transcriptional production of RNA can conceivably be limited by slow translation of RNA polymerase under N‐limitation, a fully stoichiometric view of growth should also consider transcription rates and the N → protein→RNA synthesis pathway (Hessen et al., 2007; Loladze & Elser, 2011).

In addition to elemental limitation, weakened RNA/P relationships under energy limitation have been demonstrated for biota across the tree of life (Elser et al., 2003; Rhee & Gotham, 1981), highlighting the need for a better integration of element‐energy coupling into the GRH. One way to advance this integration is by focusing on functions of ATP use in anabolic chemistry. Biosynthesis is the most CNP‐demanding and energy‐intensive process in cells (Buttgereit & Brand, 1995), meaning that even short‐term fluctuations in ATP levels could alter organismal growth‐RNA‐P coupling. After biosynthesis, ion pumps use the next highest amount of energy as they work to maintain optimal electrochemical gradients across cell membranes (Buttgereit & Brand, 1995). Thus, when an element is imbalanced across the cell membrane, cells allocate ATP to ion pumps (e.g., ATPases) to mitigate the imbalance. This activity could cause a tradeoff in ATP allocation between ion balance and transcription (≧6 ATP for transcription of a codon; Bier, 1999), or translation (~4 ATP per peptide bond; Milo & Phillips, 2015), slowing growth by decreasing the efficiency at which assimilated P is converted into biomass (Jeyasingh et al., 2020). Such costs are incurred in situations of scarcity or excess of various elements. For example, under Fe‐limitation, microbes can produce siderophores in an ATP‐intensive process (~84 ATP molecules; Hutchins et al., 1991) to bind extracellular Fe for assimilation. Note that such physiological adjustments are rarely unidimensional, as Fe‐limitation also decreases growth efficiencies via impacts on the electron transport chain (Tortell et al., 1996). On the other hand, when Fe supply is too high, cells upregulate Fe‐ATPases to reduce Fe concentration and avoid intracellular oxidative stress (e.g., Barañano et al., 2000). Overall, taking a broader perspective of growth regulation, instead of focusing largely on P, could help to understand deviations from the predictions of the GRH under different types of resource limitation.

## HOW DO WE TEST THE GRH?

### Inter‐ and intra‐specific tests of the GRH

Although the GRH was developed based on observations of aquatic invertebrates (Elser et al., 1996), because of the fundamental nature of RNA's role in driving growth, it has been widely applied to explain intra‐ and interspecific variation in biomass P content across diverse organisms (Elser et al., 2003; Godwin & Cotner, 2018; Makino et al., 2003). Here, we explain how empirical tests of the GRH can be distinguished based on their experimental design and discuss limitations and potential obstacles of applying these tests. These designs fall into three general categories of evolutionary, physiological and ontogenic comparisons, all of which are valid tests of the GRH. Combinations of tests are also valid for certain organisms, but often these designs are confounded and thus do not constitute definitive tests of the GRH, which should be kept in mind when interpreting results.

**FIGURE 3:**
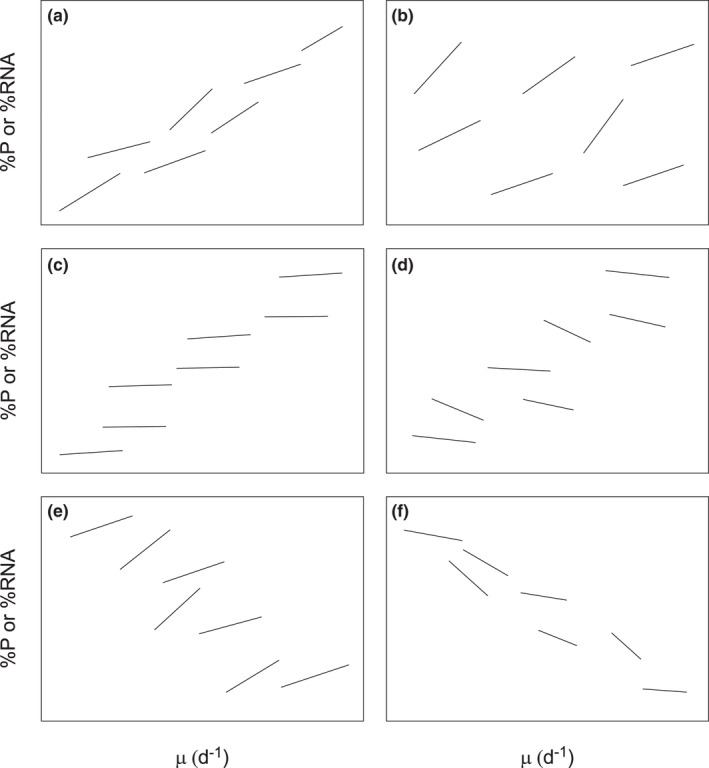
A conceptual depiction of scenarios that support or deviate from the GRH. Each line depicts a relationship between organismal growth rate (μ) and %P or %RNA. In each panel, intra‐specific data (individual lines) represent physiological and ontogenic responses within a taxon while inter‐specific patterns (comparisons of different lines) depict evolutionary differences among taxa: (a) GRH *supported* intra‐ and inter‐specifically, (b) GRH *supported* intra‐specifically but *not* inter‐specifically, (c) GRH *supported* inter‐specifically but *not* intra‐specifically (note that this is the current assumption of many stoichiometric models), (d) GRH *contradicted* intra‐specifically but *supported* inter‐specifically, (e) GRH *supported* intra‐specifically but *contradicted* inter‐specifically, and (f) GRH *contradicted* both inter‐specifically and intra‐specifically.

In *evolutionary tests*, genotypes are compared within or across species growing under similar environmental conditions and developmental stages. Inter‐specific *evolutionary tests* must consider phylogenetic context and evolutionary contingencies that have resulted in fundamental differences in the functional capability of cellular components, including the ribosome. For example, eukaryotic and prokaryotic ribosomes differ in their RNA content and especially in their protein translation rates (M3, Sterner & Elser, 2002). Indeed, typical translation rates for prokaryotic ribosomes are 17–21 amino residues per second, nearly three times higher than rates for eukaryotic ribosomes (6–9 amino residues per second, Ross & Orlowski, 1982). Thus, direct comparisons of P and RNA contents with growth rate may differ substantially across domains of life. An evolutionary test between eukaryotes and prokaryotes could fail based on these fundamental differences (Figure 3b,e or f). Additionally, genotypes within or among a species may have evolved different nutrient uptake or life‐history strategies affecting nutrient allocation patterns. Since growth is a labile trait (Lande, 2014) that can evolve in response to environmental conditions such as nutrient availability (Frisch et al., 2014; Lemmen et al., 2022a), comparisons of genotypes collected from different environments may also obscure predictions of the GRH if not taken into account.

**FIGURE 4:**
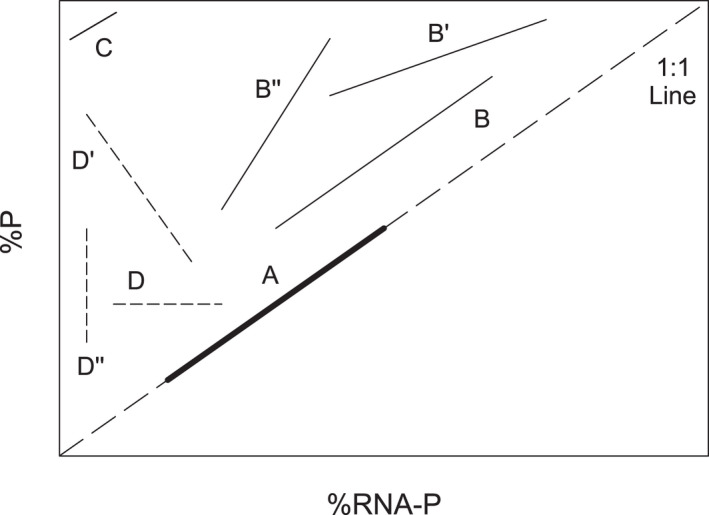
Theoretical relationships between organismal RNA and P. Relationships between organismal dry mass contributed by P found in RNA (%RNA‐P) and total organismal P content (%P) of an organism are depicted using an individual line for each species, genotype or ontogenetic stage. (A) *GRH supported* where all P is in the RNA pool (not physiologically possible), (B) *GRH supported* and RNA accounts for all variation in body %P, (B′) *GRH supported* but an organism is reallocating part its internal P pool to RNA, (B″) *GRH supported* but an organism is increasing allocation to P in other pools in addition to RNA, (C) *GRH supported* but not important (i.e., growth‐driven change in P is trivial), (D) GRH *not supported* due to shifts of stored P to RNA, (D′) GRH *not supported* because an organism is reducing its growth rate (right to left) and accumulating excess P for diapause, and (D″) GRH *not supported* due to P storage.

*Physiological tests* are tests of a single (“clonal”) genotype grown at similar developmental stages (e.g., juvenile *Daphnia)* but subjected to different experimental treatments (e.g., food quantity, quality, temperature, etc.). In these experiments, the growth rate is either manipulated directly (e.g., using chemostats) and/or indirectly by manipulating food concentrations or diet stoichiometry. Because of biological and logistical considerations, many physiological tests actually consist of comparisons of a mixed pool of sexually produced genotypes and are thus a combination of *evolutionary* and *physiological tests*. Genotype by environment experiments of these mixed populations are interesting for many reasons, but they are not rigorous tests of the GRH (which by definition operates at the *organismal* level) because the growth‐RNA‐P coupling of genotypes can vary considerably (Figure 3b). For instance, differences in the length of rDNA intergenic spacers alter the transcription rates of ribosomal genes in eukaryotes (Weider, Elser, et al., 2005) and translation (M3), growth and P retention rates in *Daphnia* across dietary P gradients (Roy Chowdhury et al., 2014). These genetic differences alter the relationship between RNA‐P and total body P contents (Figure 4B′,B″), obscuring the relationship between P and growth rate predicted by the GRH (Figure 3c). The use of clonal organisms or sexually reproducing individuals from inbred populations can be used to guard against this type of ambiguous result.

*Ontogenetic tests* are ones in which organisms are grown across their developmental cycle under similar environmental conditions. Different developmental stages of a single genotype or individual genotypes across species are compared one to another (e.g., nauplii vs. copepodites vs. adults; neonates vs. adults, etc.). *Ontogenetic tests* often involve striking changes in storage (M1), structure and metabolism of most elements, including P (Ebel et al., 2016; Sorensen, 2008). Not surprisingly, this can impact growth‐RNA‐P coupling. For example, although nutrient contents are often coupled with growth in certain plant tissues (i.e., leaves; Rivas‐Ubach et al., 2012), P‐allocation and growth rates of other plant tissues may differ throughout ontogeny, making *whole organismal* growth/nutrient relationships challenging both to quantify and interpret within the context of the GRH (Bhadra & Cai, 2019; Jing et al., 2017). When P‐allocation differs in this way, a variety of patterns could be observed, such as a stronger coupling during juvenile stages (Figure 3a or e), a weaker coupling at adult stages (Figure 3c), or even no coupling at the organismal level (Figure 3c). *Ontogenic tests* are often combined with *physiological* or *evolutionary* tests, which although not definitive tests of the GRH, can still yield useful insights. For instance, sexually reproducing copepods, *Mixodiaptomus laciniatus,* collected across field P gradients can exhibit strong GRH coupling throughout their developmental cycle despite growth rate declines of nearly 3 orders of magnitude and a reduction in RNA‐P of 60% (Bullejos et al., 2014; Carrillo et al., 2001). Total P contents also vary similarly in magnitude within and among individual life stages, and while the influence of genetic and environmental factors cannot be ruled out, %P is strongly related to biomass gain (Carrillo et al., 2001), suggesting that GRH coupling may also occur within ontogenic stages.

### Metadata analyses

Early support for the GRH first came from a synthetic study of primarily original datasets (*n* = 9), finding that, while growth/RNA and growth/P relationships differed considerably across the tree of life, these relationships within taxa tended to be positive and that RNA‐P largely accounted for variation in organismal P both within and across species (Elser et al., 2003). Considerable support for the GRH was provided by a subsequent analysis of results from 43 stoichiometrically explicit studies (predominantly consisting of datasets of P‐limited zooplankton), indicating that each of the tripartite couplings predicted by the GRH was observed ~70% of the time (Hessen et al., 2013). Other studies focusing on phytoplankton found that, while general relationships between growth rates and N:P ratios were evident (Hillebrand et al., 2013), considerable variation in phytoplankton stoichiometry/growth relationships was observed that could be attributed to P storage under non‐P limiting conditions (Flynn et al., 2010).

To update our understanding of GRH coupling across diverse taxa, we extended the original synthetic work of Elser et al. (2003) with a study of primary growth, RNA content and P content data provided by stoichiometric researchers upon request following a systematic literature search of papers referencing the “growth rate hypothesis” (see Appendix S1 for further details). We obtained data from 26 studies, containing 118 unique datasets of a single species or genotype and one dataset for a field‐collected mixed bacterial assemblage that was included in the original 2003 analysis. Most study organisms were aquatic (75%), but taxonomic coverage of the database was relatively diverse including 59 species of: zooplankton (*n* = 15 species), phytoplankton (11), aquatic invertebrates (10), terrestrial invertebrates (7), plants (8), fungi (7), bacteria (3) and human cancer cells (1). Studies consisted mostly of manipulative experiments involving P (9), P co‐limitation by other elements/macromolecules (8) or water stress (2). Two studies measured ontogenetic changes under nutrient‐replete conditions, and five studies of organisms collected from natural environments were also included.

**FIGURE 5:**
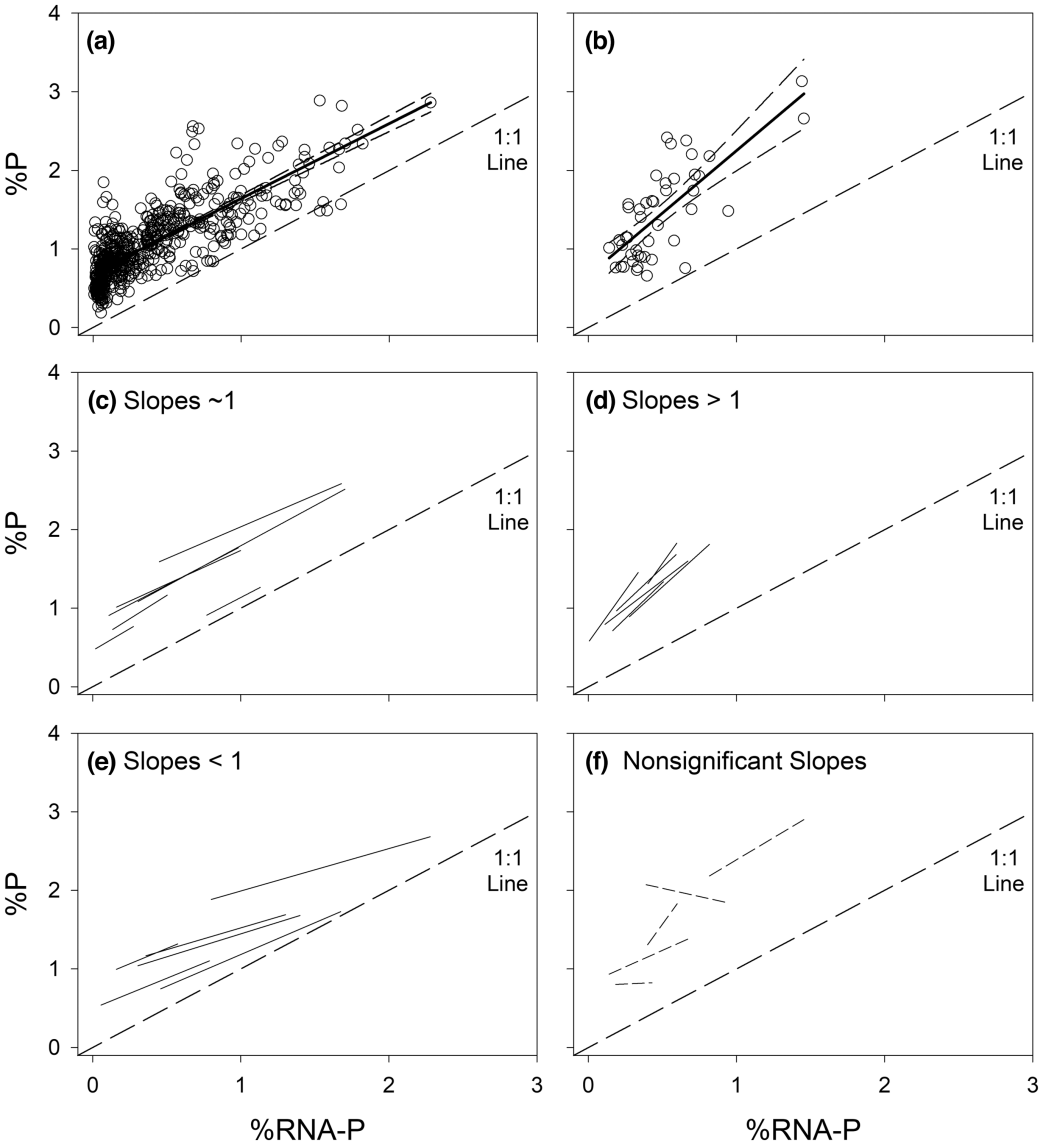
Empirical relationships between organismal %RNA‐P and total body %P. Linear regressions (dark black lines with 95% confidence intervals) across species were significant both for individual studies (a) that confirmed the GRH and (b) those that did not confirm the GRH. Reaction norms for (c) individual studies with slopes ≅ to 1 (d) >1, (e) <1 and (f) non‐significant slopes (*p* > 0.05) are also shown. Solid reaction norm lines indicate individual experiments confirming the GRH, and dotted lines are non‐confirmatory experiments. Note that realistic %RNA‐P and %P relationships are only possible above the 1:1 line.

Almost 20 years after the original evidence was presented by Elser et al. (2003), these additional data generally confirm the strong positive relationships between RNA and P predicted by the GRH. While somewhat weaker (*r* ^2^ = 0.63 compared to 0.78 in Elser et al., 2003), inter‐specific relationships between %RNA‐P and organismal body %P have remained robust in datasets confirming the GRH (*n* = 20), with a slope (0.95 ± 0.03 SE) remarkably similar to the originally reported value of 0.97 ± 0.05 SE (Figure 5). Five out of 25 datasets (20%) did not confirm predicted relationships between %RNA‐P and %P. Nevertheless, when data from these individual studies were analysed together, the combined data still confirm positive cross‐taxonomic relationships predicted by the GRH (*r* ^2^ = 0.55) with a similar slope (1.59 ± 0.22) as reported in Elser et al. (2003; 1.37). The average percentage of total biomass P found in RNA was somewhat lower than previously reported (39.6 ± 19.5 SD vs. 49 ± 5.0 *SD* in Elser et al., 2003), yet P in RNA represented a substantial, though variable, the proportion of total organismal body %P, ranging from 10.8% to 82.3% across species. Slopes of %RNA‐P to %P regressions did not differ systematically among taxa (Figure S1) but varied considerably for individual species with comparable numbers of slopes less than, greater than or indistinguishable from one. Decoupling of P from RNA was most commonly associated with non‐P‐limiting conditions, occurring under C‐limitation in ants (Kay et al., 2006) and bacteria (Elser et al., 2003) and in *Daphnia* grown under N‐limitation or nutrient‐replete conditions (Elser et al., 2003; Weider et al., 2004). Overall, these results reinforce the GRH assumption that RNA allocation in non‐vertebrates is a major factor shaping organismal body P‐content, especially under P‐limited conditions.

**FIGURE 6:**
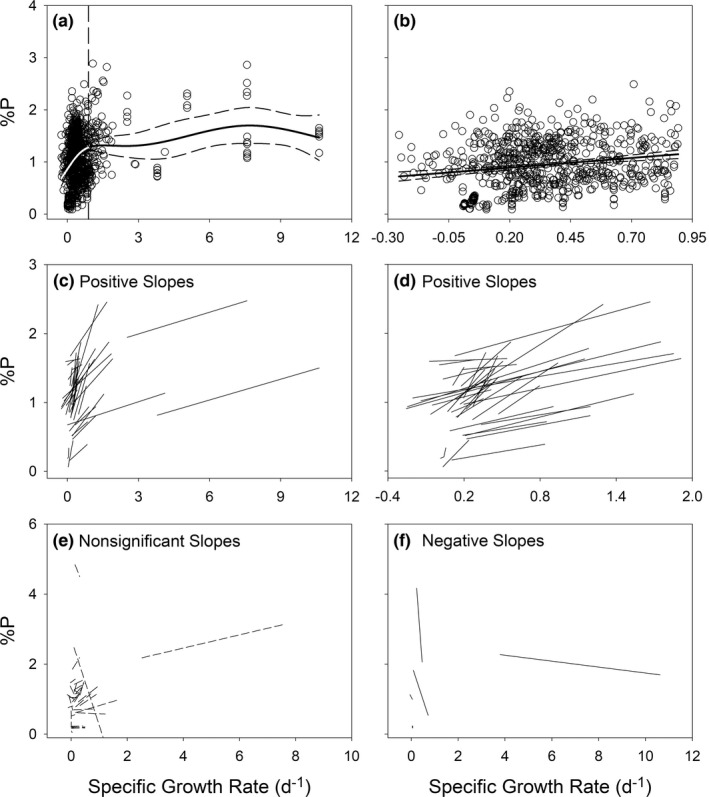
Relationships between organismal growth and body P content. Significant non‐linear relationships (*p* < 0.001; *r* ^2^ = 0.09) were found between (a) growth rates and %P across taxa using a general additive model (GAM; white and black solid line with 95% confidence intervals shown in dashed lines). Growth and %P increased linearly up to a growth threshold of 0.89 d^−1^ (dashed vertical line), identified from the first derivative of the GAM. The linear portion of the curve was also modelled separately using an ordinary linear regression in panel B (*p* < 0.001; *r* ^2^ = 0.04; slope = 0.45). Positive growth‐P reaction norms for individual datasets are shown in panel C and a subset of responses for organisms growing <2.0 d^−1^ are shown in panel D for clarity. Non‐significant (*p* > 0.05) responses are depicted in panel E, and significant negative relationships between growth and %P are shown in panel F.

In contrast to RNA and P, support for coupling between growth rate and P content was mixed, as statistically significant positive relationships were only found in 52% of datasets. Across taxa, growth was significantly (*p* < 0.001) but weakly (*r* ^2^ = 0.09) related to %P both in studies confirming the GRH (Figure 6a) and in non‐confirmatory datasets (Figure S2; *p* < 0.001, *r* ^2^ = 0.12). Linear increases in %P were observed in organisms growing up to 0.89 d^−1^, but these relationships plateaued and were insignificant above this threshold, indicating either a saturation of %RNA‐P needed to support growth or differences in RNA‐P coupling in prokaryotes at higher growth rates. Scatter around these relationships was high, and reaction norms for individual datasets were diverse, highlighting considerable taxonomic variation in how organismal P‐use is connected to growth. Indeed, we documented six cases where the growth rate was *negatively* related to %P in: photoautotrophs grown across P‐gradients or diluted growth media (Brandenburg et al., 2018; Cañavate et al., 2017), a slow‐growing detritivorous insect raised across N:P gradients (Halvorson et al., 2019), a mixed bacterial assemblage experiencing C‐limitation (Makino & Cotner, 2004), and two plants experiencing water stress (Niu et al., 2019; Sun et al., 2021). As growth‐RNA relationships across taxa were comparatively stronger than those for growth rate and %P (*r* ^2^ = 0.59; Figure S3), our analysis indicates that growth/P relationships are the weakest link in the GRH. This result is not entirely surprising as stoichiometric theory has moved from considering organismal body % P to be a fixed species‐specific property to a dynamic phenotypic trait that is better characterised by taxon‐specific reaction norms (Prater, Wagner, & Frost, 2017; Sherman et al., 2021). As GRH predictions were weakest under non‐P‐limited conditions, we now discuss the development of stoichiometric models designed to improve our understanding of mechanisms behind the uncoupling of growth, RNA and P relationships under other forms of resource limitation.

## TOWARDS NEXT‐GENERATION STOICHIOMETRIC MODELS

The development of stoichiometric models mirrors the development of the field itself, which initially focused on ecological dynamics before incorporating molecular processes. Thus, early models focused on the effects of elemental imbalances between producers and consumers on *population* growth (Andersen, 1997; Loladze et al., 2000) rather than on cellular biochemistry and *organismal* growth. These models tied producer growth to P availability, where growth decreased with producer P:C ratios until ceasing when the producer P:C ratio reached a pre‐defined minimum, (conceptually similar to the non‐ribosomal P‐pool; M1). Most importantly, food quality effects were incorporated into population dynamics theory for the first time as lower producer P:C ratios reduced consumer growth. However, consumer stoichiometry was assumed to be fixed in these models, meaning that, contrary to the GRH, reduced growth did not affect consumer P:C.

Subsequent models more directly linked consumer growth to elemental composition by including RNA and completing the tripartite coupling of the GRH. Vrede et al. (2004) showed that differences in protein:RNA ratios at maximum growth could explain macroevolutionary patterns in species N:P ratios, as originally proposed by the GRH (Elser et al., 1996; but see Seidendorf et al., 2010). Further work demonstrated that microevolutionary and ecological changes in consumer stoichiometric traits tied to growth/P coupling (e.g., rDNA intergenic spacer lengths that influence RNA transcription rates; Weider, Makino, et al., 2005; Box 1) can alter population growth dynamics and consumer P:C stoichiometry of a species (Dissanayake et al., 2019; Yamamichi et al., 2015). These models demonstrate changes in stoichiometric coupling on eco‐evolutionary scales, yet they do not model the underlying molecular processes connecting growth rate to body stoichiometry.

BOX 1 **Eco‐evolutionary implications of the GRH**

This box provides an introduction to the effects of altered biogeochemical cycling on eco‐evolutionary processes related to the GRH. Here, we chose two traits, growth and P use efficiency (PUE, biomass/P), to focus on as they are the most well‐established in the stoichiometric literature. We refer to the word “adaptation” throughout the box when we talk about adjustments or changes in behaviour, physiology and structure of an organism to become more suited to an environment (National Academies, Sciences of Engineering and Medicine, 2022).

Rapid environmental change can disproportionately alter the availability of multiple nutrients in the environment, affecting rates of organismal growth and production (Peñuelas et al., 2013; Sardans et al., 2012). Cultural eutrophication driven by anthropogenic nutrient inputs from agriculture, wastewater and urban run‐off continues to be a major problem, differentially altering N:P ratios of aquatic ecosystems (Dudgeon, 2019; Vitousek et al., 1997). Emission of N and P from fossil fuel combustion and land use change affects both aquatic and terrestrial ecosystems through atmospheric N and P deposition (Fowler et al., 2013; Scholz & Brahney, 2022; Steffen et al., 2015). This deposition can drive ecosystem shifts between N‐ and P‐ limitation even in remote regions (Elser et al., 2009, 2010; Prater et al., 2022). Fossil fuel combustion and changes in land use have also increased atmospheric carbon dioxide concentrations to unprecedented levels in human history. Since atmospheric CO_2_ equilibrates with water, aquatic ecosystems have experienced an increase of CO_2_ concentrations (Borges et al., 2006; Melack, 2016), which can alter the stoichiometric food quality and growth rates of zooplankton (Urabe et al., 2003). These perturbations impose novel selection pressures that motivate the development of eco‐evolutionary approaches to help understand and predict biotic growth responses to altered biogeochemical cycling.

Organisms can adapt to biogeochemical changes over time through evolved differences in growth rate and nutrient use efficiencies (Frisch et al., 2014; Jeyasingh et al., 2009; Lemmen et al., 2022a). For example, the rotifer *Brachionus calyciflorus* was selected for rapid growth under high P supply and developed faster growth and higher P content, consistent with the GRH (Lemmen et al., 2022b). However, rotifers selected for faster growth under P‐limitation were able to evolve faster growth rates while keeping their body P content the same. This evolutionary decoupling of the GRH can perhaps be understood via insights provided by *Daphnia* resurrection ecology experiments showing that genotypes adapted to low P conditions can have higher P retention and PUE than those adapted to high P environments (Frisch et al., 2014). As heritable genetic variation is higher for PUE than for growth and growth rate is a relatively weak predictor of *Daphnia* P content (Prater, Wagner, & Frost, 2017; Seidendorf et al., 2010), these studies indicate that divergent growth/P relationships found throughout the literature may reflect a situation in which it is not growth per se but multivariate trait evolution that shapes how organisms obtain and use elements for growth (Lande & Arnold, 1983; Sherman et al., 2017). Since growth involves more than P, this could explain unexpected, correlated reductions in minimal resource requirements and convergent evolution of biochemical/metabolic functions under differential resource limitations (i.e., N, P or energy) as observed in the green alga *Chlamydomonas reinhardtii* (Bernhardt et al., 2020; Tamminen et al., 2018).

These evolutionary adaptations of organisms can also feedback to affect ecological dynamics. For instance, *Daphnia* with higher growth rates and P‐content in high P environments (i.e., low PUE) tend to be poorer competitors under P‐limitation due to genetically mediated tradeoffs linked to metabolic (glucose phosphate isomerase; Jeyasingh et al., 2009) and ribosomal genes (rDNA; Weider, Elser, et al., 2005, Weider, Makino, et al., 2005). These tradeoffs could also explain differences in species distributions across regional food quantity and P gradients in alpine and boreal lakes (Prater, Frost, et al., 2017; Spaak et al., 2012) and may be related to habitat segregation of *Daphnia* species in hybrid complexes adapted to different aquatic environments (Tessier & Woodruff, 2002). Life‐history evolution might also drive ecological dynamics such as eutrophication‐induced community composition shifts from highly efficient, slow‐growing, low P (i.e., high PUE) taxa to fast‐growing, less efficient, high P taxa with nutrient enrichment in streams (Gafner & Robinson, 2007; Singer & Battin, 2007). However, growth and body stoichiometry of individual species can often be poor predictors of species‐specific shifts or community biomass stoichiometry (Beck et al., 2021; Evans‐White et al., 2009), complicating long‐standing predictions of consumer‐driven nutrient cycling (Sterner et al., 1992).

Despite its focus on P and RNA, the growth rate limiting mechanism of the GRH is translation, the synthesis of proteins from N‐rich amino acids. This protein‐centric view has traditionally been the norm in microbial growth research, with one class of ribosome models that even treats ribosomes as self‐replicating entities made entirely of protein (Koch, 1988; but also see Klumpp et al., 2013; Scott et al., 2014). These simple models can explain linear relationships between ribosome content and growth rate through cellular optimisation of translational capacity by differential expression of ribosomal and non‐ribosomal proteins. Ribosomal P costs play no role in these models because their formulations do not include RNA, and maximal growth rates should occur at 100% ribosomal protein production, which is biologically impossible. To avoid this problem of “unlimited ribosomes,” these models constrain ribosomal abundance with a fixed, growth‐invariant “overhead” fraction of non‐ribosomal proteins (Scott & Hwa, 2011). A more biologically meaningful way of incorporating ribosomal production costs into growth models is to focus on the differential N and P demands associated with ribosomal proteins and RNA (Kafri et al., 2016).

In order to grow, organisms must first acquire N and P from the environment to produce ribosomal proteins and RNA. A stoichiometric model dynamically linking translation and transcription shows that, for any given N:P supply ratio, there exists a unique translation:transcription ratio and corresponding organismal N:P ratio associated with balanced growth (i.e., where all major biochemical pools grow at the same rate; Loladze & Elser, 2011). This model shows that balanced microbial growth occurs at N:P supply ratios near Redfield proportions of 16:1 (Redfield, 1934), and microbial N:P ratios themselves are 16:1 under nutrient‐replete conditions due to the balance of translation and transcription. Limitation by either element can reduce these rates (Kafri et al., 2016), altering relationships between microbial growth rate, protein:RNA ratios and N:P ratios. Specifically, at N:P supply ratios above 16:1 growth is P‐limited leading to reduced RNA transcription rates, whereas low N:P supply ratios reduce growth due to N‐limitation of protein translation. In either case, microbial N:P ratios for balanced growth deviate from 16:1 and instead fall between this ratio and the N:P supply ratio (Loladze, 2019). Thus, for a more complete stoichiometric understanding of growth limitation under imbalanced resource supplies, transcription and translation should be given equal consideration in stoichiometric growth models.

While the above models are individually capable of addressing certain GRH weakening mechanisms (M1 and M3), further extensions to growth models provide a more comprehensive view of stoichiometric growth physiology. Using a conceptually similar model formulation as Loladze and Elser (2011), Li et al. (2018) constructed a dynamical system to examine how changes in ribosome activity (M2) and protein elongation rate (M3) under C‐, N‐ and P‐limitation mediate population growth rates μ of *E. coli*. Like most growth models, μ is defined as the relative increase in protein mass, which is described by the equation

$$\mu =\frac{R_{w}k_{\mathrm{el}}m_{a}}{P_{m}}.$$

$R_{w}k_{\mathrm{el}}m_{a}$ is the mass of protein produced by active ribosomes, $R_{w}$, where $k_{\mathrm{el}}$ is the peptide chain elongation rate, $m_{a}$ is the average mass of amino acid and $P_{m}$ is the total protein mass in a cell. While not originally formulated as a stoichiometrically explicit model, Phan et al. (2021) recently connected the Li et al. (2018) model to the GRH by calculating bacterial N:P ratios under each form of limitation, finding good agreement with empirical measurements. Further, they showed that this model is capable of coherently capturing all experimental observations under different nutrient limitation scenarios, providing a powerful framework for identifying physiological mechanisms responsible for weakening GRH coupling under different forms of nutrient limitation.

To include protein degradation effects (M4) in this model, degradation rates could easily be introduced into the rate of change of the protein pool ($d\left(P_{m}\right)/\mathrm{dt}$). Incorporating the effects of non‐RNA P‐storage (M1), however, is more complex. One underlying assumption in the growth rate expression is that the ratios of protein mass ($P_{m}$) and production ($R_{w}k_{\mathrm{el}}m_{a}$) to total biomass (F) and production of all other biomolecules (f) are constant $\left(c\right)$ at steady state or balanced growth. That is

$$\begin{array}{c} \mu =\frac{R_{w}k_{\mathrm{el}}m_{a}+f}{P_{m}+F}=\frac{R_{w}k_{\mathrm{el}}m_{a}+c\left(R_{w}k_{\mathrm{el}}m_{a}\right)}{P_{m}+cP_{m}}\\ \;=\frac{R_{w}k_{\mathrm{el}}m_{a}\left(1+c\right)}{P_{m}\left(1+c\right)}=\frac{R_{w}k_{\mathrm{el}}m_{a}}{P_{m}}. \end{array}$$

To account for M1, we can omit the constant ratio assumption and explicitly introduce the dynamics of non‐RNA P storage into variables f and F to study their relationship with growth rate. These two parameters could also be used to incorporate energy costs into this framework (Phan et al., 2021) and to form a comprehensive stoichiometric growth model by considering C pools, including polyesters (Poblete‐Castro et al., 2012), carbohydrates (Liefer et al., 2019), and lipids (Wagner et al., 2015). Such C‐rich molecules can represent a significant proportion (20%–80%) of total biomass under N‐ and/or P limitation, so their inclusion would allow stoichiometric models to better predict C:P and C:N ratios in addition to N:P.

This model formulation can also be used to address some discontinuities between how the GRH is conceptualised, modelled and tested. Perhaps the greatest conceptual divide in GRH research is that, while growth rate is most often empirically measured as the mass‐specific rate of total biomass production, it is theoretically and mathematically considered in terms of specific protein production. These inconsistencies can be addressed by relaxing the constant ratio assumption and expressing the active ribosomal pool and protein mass and production rates relative to total biomass, making these estimates more comparable to empirical measurements. Other dynamics not yet explored using this approach include the effects of transcription and mechanisms M1‐M4 on stoichiometric coupling during imbalanced growth. This knowledge is crucial for formulating GRH‐based predictions under environmentally relevant scenarios where variation in environmental N:P supplies could alter organismal growth‐RNA‐P coupling and for integrating these physiological responses into existing stoichiometric population dynamics models.

Together, these modelling efforts have greatly increased our stoichiometric understanding of growth, and their extensions may be used to test the applicability and limits of the GRH. So, what would these extended models entail? Optimally, they should: (1) express organismal growth rates as the translational output of the active ribosomal pool relative to total organismal biomass, (2) limit ribosomal biogenesis by transcription/translational rates and their associated elemental and energetic constraints and (3) explicitly consider mechanisms M1‐M4, which directly affect GRH coupling. A comprehensive model of the GRH should be able to dynamically connect stoichiometric nutrient availability to the macromolecular pools and physiological rates that control organismal C:N:P ratios and growth. This model should also be flexible enough to study GRH dynamics in eco‐evolutionary, physiological and ontogenic contexts to enable integrative collaborative research efforts between modellers and empiricists.

## CONCLUSIONS: TOWARDS A HOLISTIC UNDERSTANDING OF GROWTH

When theory conflicts with sound empirical measurements, theoretical revisions are in order. Experimental and modelling efforts over two decades suggest that this is the case for the GRH, as it is for other aspects of stoichiometric theory. By focusing on four key mechanisms that directly shape growth, biochemical and stoichiometric coupling, we have provided a conceptual framework for generating a better understanding of growth using stoichiometric principles. The couplings posited by the GRH (and decoupling discussed herein) are established by the intense cellular interplay among key non‐substitutable resources (energy, C, N and P) that are connected by core metabolic processes common to all organisms—ATP generation, ribosome production, protein synthesis and mass/energy storage.

To develop a more robust GRH that advances the field of biological stoichiometry, a broader range of rigorous, cross‐taxon tests of the connections among growth, macromolecular composition and C:N:P stoichiometry are needed. The most useful, and challenging, advances will come from combining these more standard measurements with those of key cellular processes of ribosome activity and protein production and turnover rates. Without this knowledge, we lack fundamental information for formulating predictive models of growth based on first principles that include the law of mass conservation and the central dogma of molecular biology. To achieve this goal, closely integrated physiological, evolutionary and ecological experiments across model organisms that represent key functions in ecosystems and within the framework of stoichiometric theory are necessary. We also need more studies of environmental effects on the GRH in natural environments, especially for abundant organisms that grow well under low elemental and energy supplies that are common in the field. Incorporation of non‐RNA P into the GRH framework could allow for the inclusion of long‐exiled taxa (i.e., large organisms and vertebrates), providing fresh insights by broadening the taxonomic scope of the field. It could be argued that the applicability of the GRH has been limited by its predominant focus on P; if so, the more diverse and inclusive framework presented here should ensure that the GRH will contribute substancially towards the ongoing advancements in our predictive understanding of growth.

## AUTHOR CONTRIBUTIONS

All authors conceptualized the study. JIN, CP, and JJE wrote the manuscript with contributions from all co‐authors. CP and JIN conducted the meta‐analysis.

### PEER REVIEW

The peer review history for this article is available at: https://publons.com/publon/10.1111/ele.14096.

## Supporting information


**Appendix S1** Supporting Information

## Data Availability

No new data were used in this study. Data supporting our results are archived at Figshare (10.6084/m9.figshare.20493162).
