# Supplementary material for: Revisiting the growth rate hypothesis: Towards a holistic stoichiometric understanding of growth
Source: Ecol Lett. 2022 Sep 11;25(10):2324–39. doi: 10.1111/ele.14096 (PMC9595043; doi:10.1111/ele.14096)
Supplement: Supplementary file 1 — Appendix S1 Supporting Information [file ELE-25-2324-s001.docx]

**Supporting Information S1**

**Revisiting the Growth Rate Hypothesis: towards a holistic stoichiometric understanding of growth**

Jana Isanta-Navarro, Clay Prater, Logan M. Peoples, Irakli Loladze, Tin Phan, Punidan D. Jeyasingh, Matthew J. Church, Yang Kuang, and James J. Elser

**Metadata Analysis**

*Methods and Database description*

To examine the strength of predicted coupling among growth, RNA content, and P content proposed by the growth rate hypothesis (GRH) across a diverse range of organisms, we conducted a systematic search for manuscripts containing the search term “growth rate hypothesis” in Google Scholar on October 21, 2021, resulting in 2,340 hits. Google Scholar limits the result to the 1,000 most relevant papers, that mentioned the “growth rate hypothesis”. Our search was systematic but not exhaustive; however, other sources such as Scopus yielded fewer hits than our initial 1,000 papers. We evaluated all 1,000 papers and found 179 potential studies representing a wide range of study taxa and publication dates 1991-2021. For inclusion, we required that a study measure at least 2 out of 3 GRH parameters (P-content, RNA-content, and growth). To make our results comparable to the original GRH metadata analysis (Elser *et al.* 2003), we also required that studies reported specific growth rates μ d^-1^ [ln ((initial measurement)/final measurement)/time)] and that RNA and P were expressed as percent dry mass values. Studies with biologically impossible values where %RNA-P was higher than the total P of an organism were also excluded, leaving us with a final pool of 129 suitable manuscripts. Archived datasets were downloaded when available, but most data were generously provided by researchers following requests. Primary datasets for a total of 26 studies (20% of requested datasets) were used in our metadata analysis (Supplementary Table 1).

Prior to analysis, growth measurements were Q_10_ -corrected to 20°C, and for the few studies that directly manipulated temperature, we only included one treatment closest to 20°C to avoid confounding comparisons to all other studies. Individual datasets were divided into two groups: those confirming the positive tripartite linkages predicted by the GRH (i.e. positive correlations with a P-value below 0.05; 50.4% of total datasets; hereafter referred to as confirmatory) or nonconfirmatory studies showing either significant negative or nonsignificant linear relationships (49.6%; *P* > 0.05).

All linear ordinary least squares regressions were performed in either Microsoft Excel (Redmond, Wa) for comparability to the original Elser et al. (2003) analysis and/or SigmaPlot (Systat, Palo Alto, Ca) to generate reaction norm plots. Nonlinear relationships between growth, RNA, and %P were analyzed across taxa in confirmatory and nonconfirmatory datasets separately using general additive models, using the R-packages “mgcv” and “gratia” (Wood 2011; Simpson 2018). Significant changes in each relationship were determined using the first derivative method according to Simpson (2018). As our final dataset contained data from Elser et al. (2003), we consider this work an extension of the previous work. Nevertheless, to ensure that these data did not bias our current interpretations, all comparable statistics were ran with and without the original data, and where cross comparisons are made in the text, data from the original paper were excluded

Literature cited:

Elser, J.J., Acharya, K., Kyle, M., Cotner, J., Makino, W., Markow, T., et al. (2003). Growth rate-stoichiometry couplings in diverse biota. Ecol. Lett., 6, 936–943.

Simpson, G.L. (2018). Modelling Palaeoecological Time Series Using Generalised Additive Models, Front. Ecol. Evol., 6, 149.

Wood, S.N. (2011). Fast stable restricted maximum likelihood and marginal likelihood estimation of semiparametric generalized linear models. J. R. Stat. Soc. Ser. B Stat. Methodol., 73, 3–36.

Supporting Table 1: Manuscripts included in the metadata analysis.

| Authors | Year Published | Journal | DOI |
| --- | --- | --- | --- |
| Agren & Weih | 2012 | New Phytologist | [10.1111/j.1469-8137.2012.04114.x](applewebdata://82C74CBC-75FB-408F-A78E-E162EDB373C9/10.1111/j.1469-8137.2012.04114.x) |
| Balseiro et al. | 2021 | Scientific Reports | [10.1038/s41598-021-91959-w](applewebdata://82C74CBC-75FB-408F-A78E-E162EDB373C9/10.1038/s41598-021-91959-w) |
| Brandenburg et al. | 2018 | Ecology Letters | [10.1111/ele.13138](applewebdata://82C74CBC-75FB-408F-A78E-E162EDB373C9/10.1111/ele.13138) |
| Buellejos et al. | 2014 | Plos One | [10.1371/journal.pone.0086493](https://doi.org/10.1371/journal.pone.0086493) |
| Cañavate et al. | 2017 | Journal of Plant Physiology | [10.1016/j.jplph.2017.03.019](applewebdata://82C74CBC-75FB-408F-A78E-E162EDB373C9/10.1016/j.jplph.2017.03.019) |
| Elser et al. | 2003 | Ecology Letters | [10.1046/j.1461-0248.2003.00518.x](https://doi.org/10.1046/j.1461-0248.2003.00518.x) |
| Elser et al. | 2006 | Functional Ecology | [10.1111/j.1365-2435.2006.01165.x](https://doi.org/10.1111/j.1365-2435.2006.01165.x) |
| Elser et al. | 2007 | Plos One | [10.1371/journal.pone.0001028](https://doi.org/10.1371/journal.pone.0001028) |
| Ferrão-Filho et al. | 2005 | Freshwater Biology | [10.1111/j.1365-2427.2005.01378.x](http://dx.doi.org/10.1111/j.1365-2427.2005.01378.x) |
| Ferrão-Filho et al. | 2007 | Limnology and Oceanography | [10.4319/lo.2007.52.1.0407](https://doi.org/10.4319/lo.2007.52.1.0407) |
| González et al. | 2013 | Freshwater Biology | [10.1111/fwb.12300](https://doi.org/10.1111/fwb.12300) |
| Grimmeet et al. | 2015 | Fungal Ecology | [10.1016/j.funeco.2013.08.002](https://doi.org/10.1016/j.funeco.2013.08.002) |
| Gulis et al. | 2017 | ISME Journal | [10.1038/ismej.2017.123](applewebdata://82C74CBC-75FB-408F-A78E-E162EDB373C9/10.1038/ismej.2017.123) |
| Halvorson et al. | 2019 | Oecologia | [10.1007/s00442-019-04409-w](applewebdata://82C74CBC-75FB-408F-A78E-E162EDB373C9/10.1007/s00442-019-04409-w) |
| Hessen et al. | 2007 | Functional Ecology | [10.1111/j.1365-2435.2007.01306.x](https://doi.org/10.1111/j.1365-2435.2007.01306.x) |
| Hood & Sterner | 2014 | Functional Ecology | [10.1111/1365-2435.12243](https://doi.org/10.1111/1365-2435.12243) |
| Hu et al. | 2020 | Plant Biology | [10.1111/plb.13111](applewebdata://82C74CBC-75FB-408F-A78E-E162EDB373C9/10.1111/plb.13111) |
| Kay et al. | 2006 | Functional Ecology | [10.1111/j.1365-2435.2006.01187.x](https://doi.org/10.1111/j.1365-2435.2006.01187.x) |
| Lukas et al. | 2011 | Functional Ecology | [10.1111/j.1365-2435.2011.01876.x](https://doi.org/10.1111/j.1365-2435.2011.01876.x) |
| Niu et al. | 2019 | Plant Biology | [10.1111/plb.12897](https://doi.org/10.1111/plb.12897) |
| Prater et al. | 2017 | Ecology | [10.1002/ecy.1795](https://doi.org/10.1002/ecy.1795) |
| Shimizu & Urabe | 2008 | Oecologia | [10.1007/s00442-007-0896-7](applewebdata://82C74CBC-75FB-408F-A78E-E162EDB373C9/10.1007/s00442-007-0896-7) |
| Spijkerman & Wacker | 2011 | Extremophiles | [10.1007/s00792-011-0390-3](applewebdata://82C74CBC-75FB-408F-A78E-E162EDB373C9/10.1007/s00792-011-0390-3) |
| Sun et al. | 2021 | Global Ecology and Conservation | [10.1016/j.gecco.2020.e01416](https://doi.org/10.1016/j.gecco.2020.e01416) |
| Valdivia-Anistro | 2016 | Frontiers in Microbiology | [10.3389/fmicb.2015.01486](https://doi.org/10.3389/fmicb.2015.01486) |
| Weider et al. | 2004 | Limnology and Oceanography | [10.4319/lo.2004.49.4_part_2.1417](https://doi.org/10.4319/lo.2004.49.4_part_2.1417) |


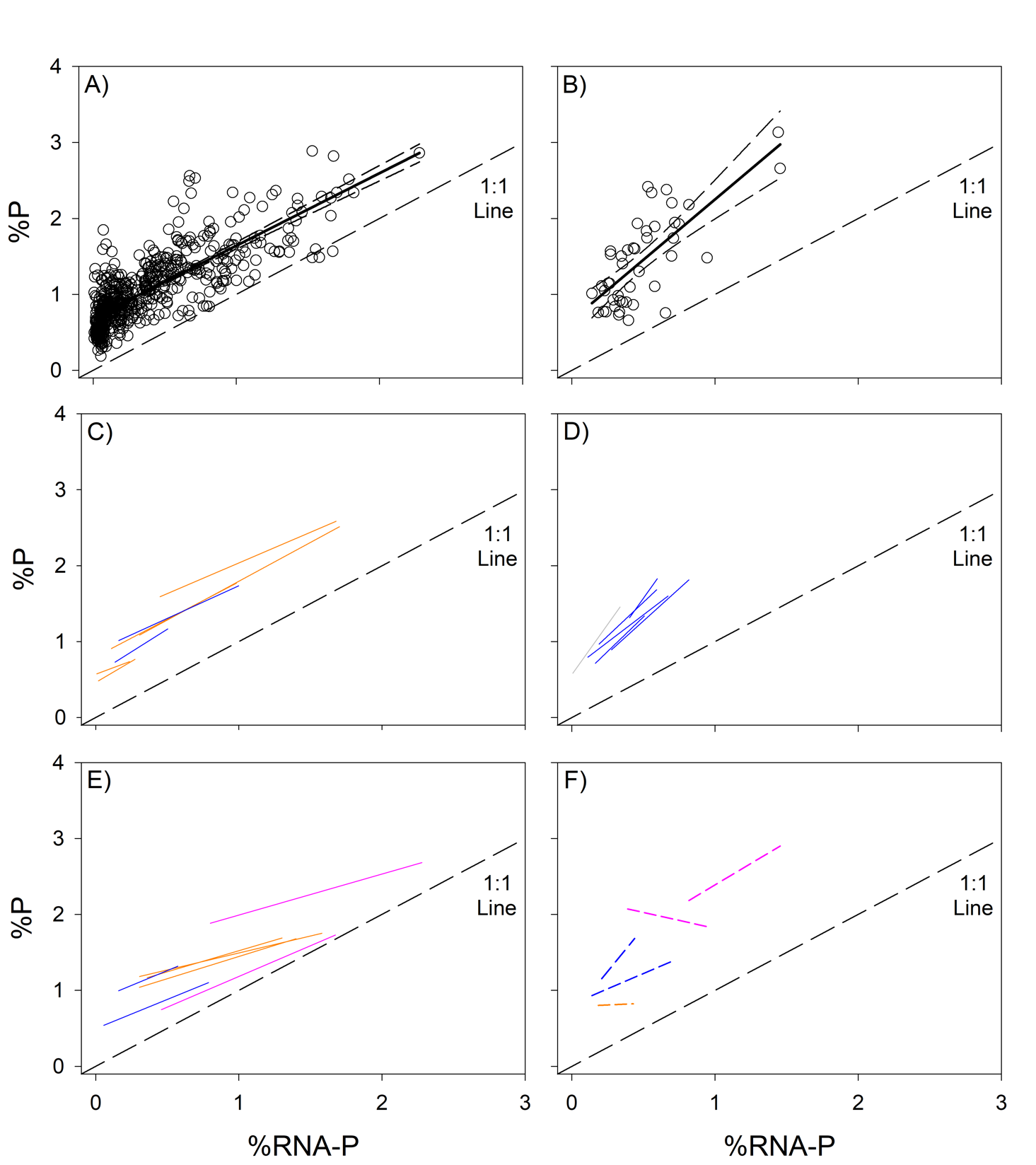
Supporting Figure 1: Taxonomic difference in relationships between organismal %RNA-P and total body %P. Linear regressions (dark black lines with 95% confidence intervals) across species were significant both for individual studies (A) that confirmed the GRH and (B) those that did not confirm the GRH. Reaction norms for C) individual studies with slopes ≅ to 1 D) >1, E) <1, and F) non-significant slopes (*P* >0.05) are also shown. Solid reaction norm lines indicate individual experiments confirming the GRH, and dotted lines are non-confirmatory experiments. Line colors denote taxonomic groups including: bacteria (pink), zooplankton (blue), terrestrial invertebrates (orange) and human cancer cells (gray) Note that realistic %RNA-P and %P relationships are only possible above the 1:1 line.

Supporting Figure 2: Relationships between organismal growth and body phosphorus (P)-content in studies not supporting predictions of the GRH. A significant non-linear relationship (*P*<0.001; r^2^= 0.12) was found between specific growth rate and %P across taxa using a general additive model (GAM; white and black solid line with 95% confidence intervals shown in dashed lines). Growth and %P changed non-linearly up to a growth threshold of 0.61 d^-1^ (dashed vertical line), identified from the first-derivative of the GAM.

Supporting Figure 3: Relationships between organismal growth and body phosphorus RNA-content. A significant non-linear relationship (*P*<0.001; r^2^= 0.59 was found between specific growth rate and %RNA across taxa using a general additive model (GAM; white and black solid line with 95% confidence intervals shown in dashed lines). Growth and %RNA changed non-linearly between 0.13-1.22 and 3.9- 5.8 d^-1^ (dashed vertical lines), identified from the first-derivative of the GAM.
